# Amperometric biosensing system directly powered by button cell battery for lactate

**DOI:** 10.1371/journal.pone.0212943

**Published:** 2019-03-06

**Authors:** Xiaojin Luo, Xuesong Yao, Yalei Zhang, Xingwen Zheng, Guangming Xie, Yue Cui

**Affiliations:** 1 Department of Materials Science and Engineering, College of Engineering, Peking University, Beijing, China; 2 The State Key Laboratory of Turbulence and Complex Systems, College of Engineering, Peking University, Beijing, China; 3 Insititute of Ocean Research, Peking University, Beijing, China; Beijing Institute of Technology, CHINA

## Abstract

The development of new signal systems for electrical biosensors could provide exciting new opportunities for biomedical analysis, pollutant monitoring, and explosive detection. The signal systems for commercial portable sensors involve the integration of a battery and a circuit conditioning system to power an amperometric biosensor. However, this increases the size and complexity of the entire system. In this study, we develop a simple amperometric biosensor that is directly powered by a button cell battery for the detection of lactate. A two-electrode sensing transducer was printed on cardboard or integrated on a ring. It was directly powered by a button cell battery, and connected to a multimeter for current measurement. This sensor showed a sensitive detection range of 0.04762–9.21429 mM and short measuring time of 2 min. These results show that this system can achieve an excellent sensing performance, and the construction of this new sensing system directly powered by a button cell battery offers a new method for further developing a wide range of miniaturized, flexible, portable, or wearable sensing systems, and these could be used in detecting various analytes that are important in medical diagnosis and environmental monitoring.

## 1. Introduction

Amperometric biosensing systems have been widely used for healthcare applications [1–3]. In such sensors, the sensing transducer consists of three electrodes: working, reference, and counter electrodes [1–3]. The sensor configuration could have three or two electrodes, a bioreceptor is generally immobilized on the working electrode, and all electrodes are covered by buffer solution [4–6]. In a two-electrode sensor, one electrode functions as both the reference and the counter electrodes [6]. The sensor is generally powered by a signal system consisting of a battery and a circuit conditioning system [1, 2]. The latter regulates the battery voltage to produce a suitable output power for the sensor. Currently, micrometer or millimeter-scale sensing transducers can be fabricated or printed [1, 6]. However, the entire sensing system cannot be too small owing to the size of the battery and circuit conditioning system.

Miniaturizing the entire biosensing system is important for realizing applications based on flexible biosensors, wearable biosensors, and Internet of things [1, 2, 7, 8]. Recently, studies have tried to develop novel miniaturized sensors with an integrated battery, regulator, circuit conditioning system, and substrate [1–3, 7–12]. However, the developed system still has a relatively large size, which generally has a benchtop size. Lactate is always used as a target analyte for developing these systems [1, 2], since it is an important metabolic analyte in human eccrine sweat and is related to sports medicine [13–15].

The present study is the first to report a button cell battery with a constant output voltage of 1.5 V for directly powering an amperometric biosensor for lactate detection. To reduce the current baseline level, a 2 mM buffer is used instead of the conventional 50 or 100 mM buffer. The sensor is constructed by printing carbon graphite ink as a working electrode and Ag/AgCl ink as a reference/counter electrode on a hard paper or on a ring to form a two-electrode sensing transducer. The cathode and anode of the battery are connected to the two electrodes of the sensing transducer. Lactate oxidation is catalyzed by the immobilized lactate oxidase on the working electrode to generate hydrogen peroxide (H_2_O_2_). H_2_O_2_ is further oxidized at the electrode to generate a current signal that is measured by a current meter.

We show here for the first time that a simplified circuit in amperometric biosensing system directly powered by button cell battery has an excellent sensing performance, rather than a complex and large instrument such as a potentiostat [5, 16, 17]. This new system could further minimize the size of the entire sensing system and lower the cost, since it employs a battery to power the sensor directly without using a complex circuit in between. This offers a new approach for the construction of small sensing systems that are important for the development of various, new flexible, wearable, or portable sensing systems [5, 16, 17]. These miniaturized, flexible, wearable, or portable systems could advance the sensing approaches for detecting a variety of analytes in different samples, such as blood, sweat, wastewater, and this could advance various medical and environmental applications.

We first introduce the optical characterization of the sensor setup, followed by the optimization of the voltage and the buffer concentration for the sensor. Then, we compare a potentiostat and a button cell battery for the detection of H_2_O_2_ and lactate, to see whether a button cell battery can result in similar sensing performances as a potentiostat.

## 2. Experimental

### 2.1. Apparatus and chemicals

A digital multimeter model 34465A with BenchVue software was purchased from Keysight (Santa Rosa, CA, USA). A potentiostat CHI660e was obtained from CH Instruments, Inc. (Shanghai, China). An optical microscope was acquired from Cewei, Inc. (Shanghai, China). Button cell batteries were acquired from Renata, Inc. (Itingen, Switzerland). A 3D printer model SLA550 was obtained from ZRapid Technology Inc. (Suzhou China). Carbon graphite and Ag/AgCl inks were obtained from Gwent Electronic Materials Ltd. (Pontypool, UK). Glutaraldehyde and sodium L-lactate were purchased from Sigma-Aldrich Inc. (Beijing, China). Sodium hydroxide was acquired from Beijing Chemical Works (Beijing, China). Sodium dihydrogen phosphate was purchased from Tianjin Fuchen Chemical Reagent Factory (Tianjing, China). Hydrogen peroxide was purchased from Beijing Chemical Works (Beijing, China).

### 2.2. Sensing electrode preparation

A cardboard was cut into a piece with area of 2.5-cm width and 4-cm length. A tape was used to cover the electrode boundary with 15-mm length and 2-mm width; the top side was coated with the graphite paste as the working electrode and the bottom side was coated with the Ag/AgCl paste as the reference electrode. After finishing, the sensor was placed in an oven at 80°C for 30 min. Then, the adhesive tape was torn off, and the heated epoxy was spread on one end of the electrode to create a space with a 1-cm width, a 2-cm length, and a 0.5-cm height. A 2 mm × 2 mm area for each electrode was used as the sensing part.

For the ring-based sensor, a hollow ring was printed by a 3D printer using a photosensitive resin material with 8-mm outer radius, 5-mm inner radius, and 5-mm height. The battery receiving hole inside the ring is a cylindrical space with 1.8-mm width and 4.8-mm radius. A button cell battery for powering the sensor was embeded into the ring. The carbon graphite and Ag/AgCl electrodes were manually painted on the inner side of the ring. Each electrode has a width of 3 mm and a length of 2 mm, with a space of 1 mm in between.

### 2.3. Enzyme immobilization

A 2 μl mixture of lactate oxidase (10 U μl^-1^) and 2% glutaraldehyde was vigorously shaken and dripped onto the working electrode with a pipette. Then, the sensor was placed in a refrigerator at 4°C overnight. The next day, the sensor was removed, the electrode was covered with a buffer solution for 1 h, and measurements were started.

### 2.4. Biosensor connection to battery

The positive electrode of a battery case with some button batteries was connected with the graphite electrode of the sensor using copper paper, negative electrode of the battery case was connected to the negative probe of the multimeter using a clip, and positive probe of the multimeter was connected to the Ag/AgCl electrode of the sensor using a clip.

### 2.5. Sensing measurements

A Keysight multimeter connected to a computer running a BenchVue software was used for the sensing measurements with a battery at a room temperature of 24°C. The DC measurement mode was used for current measurements, with the cardboard sensor being directly powered by a button cell battery. A potentiostat was used for the amperometric measurement of the sensor at a constant voltage as well.

## 3. Results and discussions

### 3.1. Optical characterization of the sensor

**Fig 1:**
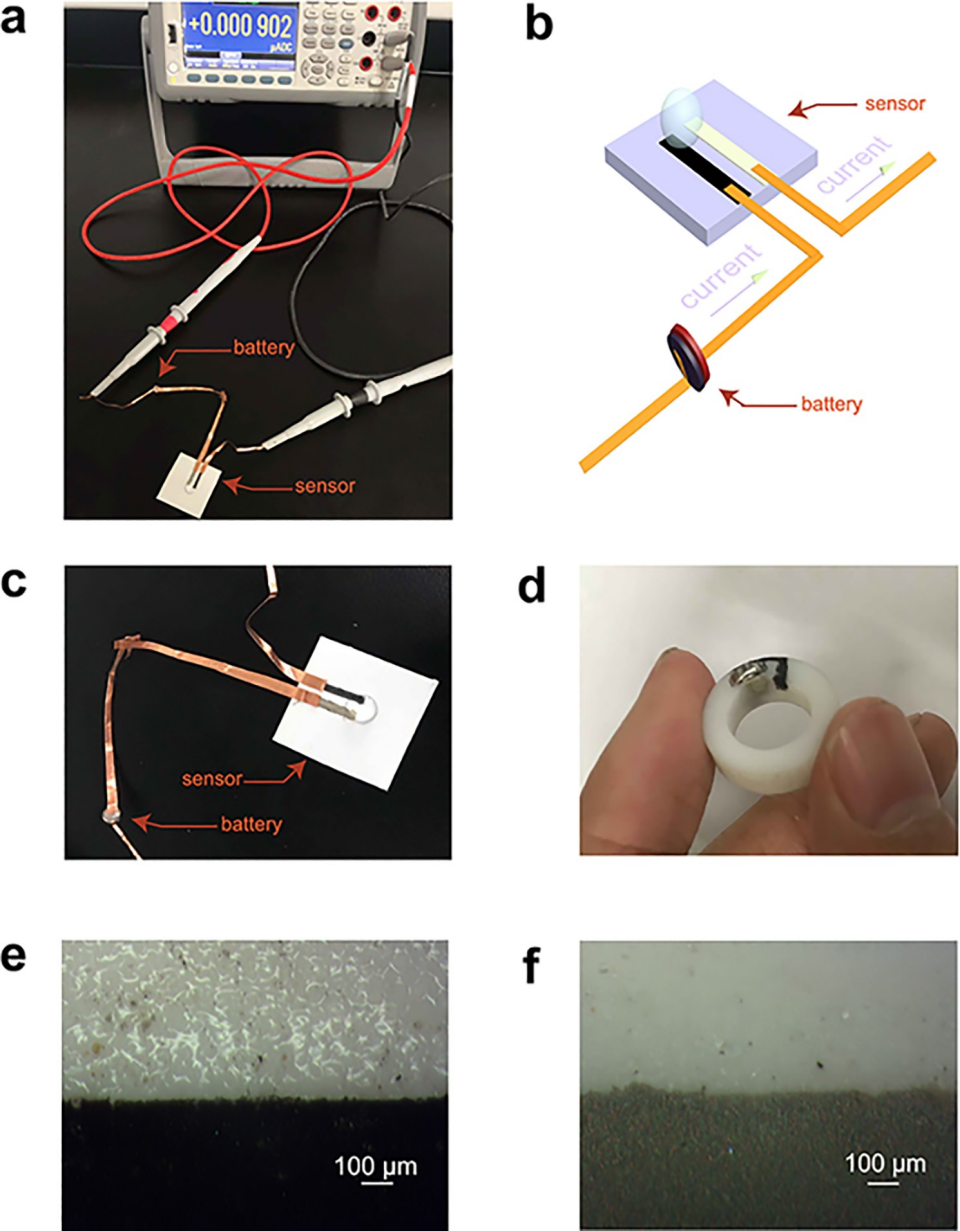
Images of the sensor powered by a button cell battery. (a) A photograph of the experimental setup for the sensing sytem. (b) A schematic illustration of the connection between a button cell battery and a sensor. (c) A camera image of the sensor printed on cardboard. (d) A camera image of the sensor and a battery system embedded into a 3D-printed ring. (e) An optical image of a carbon-graphite electrode on the sensor. (f) An optical image of Ag/AgCl electrode on the sensor.

Fig 1 shows the photographs and optical images of the sensing systems. Fig 1A shows a photograph of the experimental setup for the sensing sytem, including the circuit structure with the connection of a button cell battery, a multimeter and a sensing electrode. Fig 1B shows a schematic illustration of the connection between a button cell battery and a sensor. As shown in Fig 1B, the positive terminal of the button battery is connected with the working electrode of the sensor by the copper wire, and the negative terminal is connected with the negative probe from the multimeter by wire, and the positive probe from the multimeter is connected with the opposite electrode of the sensor. Fig 1C shows the camera image of the cardboard-based sensor with the battery used for measurements in this study. The graphite electrode in a black color is the working electrode, and the Ag/AgCl electrode in a gray color is the reference electrode. The distance between the two electrodes is 2 mm, the length of each electrode is 15 mm, and the width of each electrode is and 2 mm. When using a potentiostat, the potentiostat supplies a stable potential between the two electrodes. When using a battery as the power source, the battery directly supplies a voltage between the two electrodes, and current signals were measured using a multimeter. Fig 1D shows a battery-powered ring sensor that can be worn on the finger to detect lactate in sweat. Fig 1E shows a graph of the graphite electrode and Fig 1F, an optical image of the Ag/AgCl electrode. The results demonstrate that the sensing electrodes can be constructed successfully and can then be connected to the power system.

### 3.2. Optimization of voltage and buffer concentration for the sensing system

**Fig 2:**
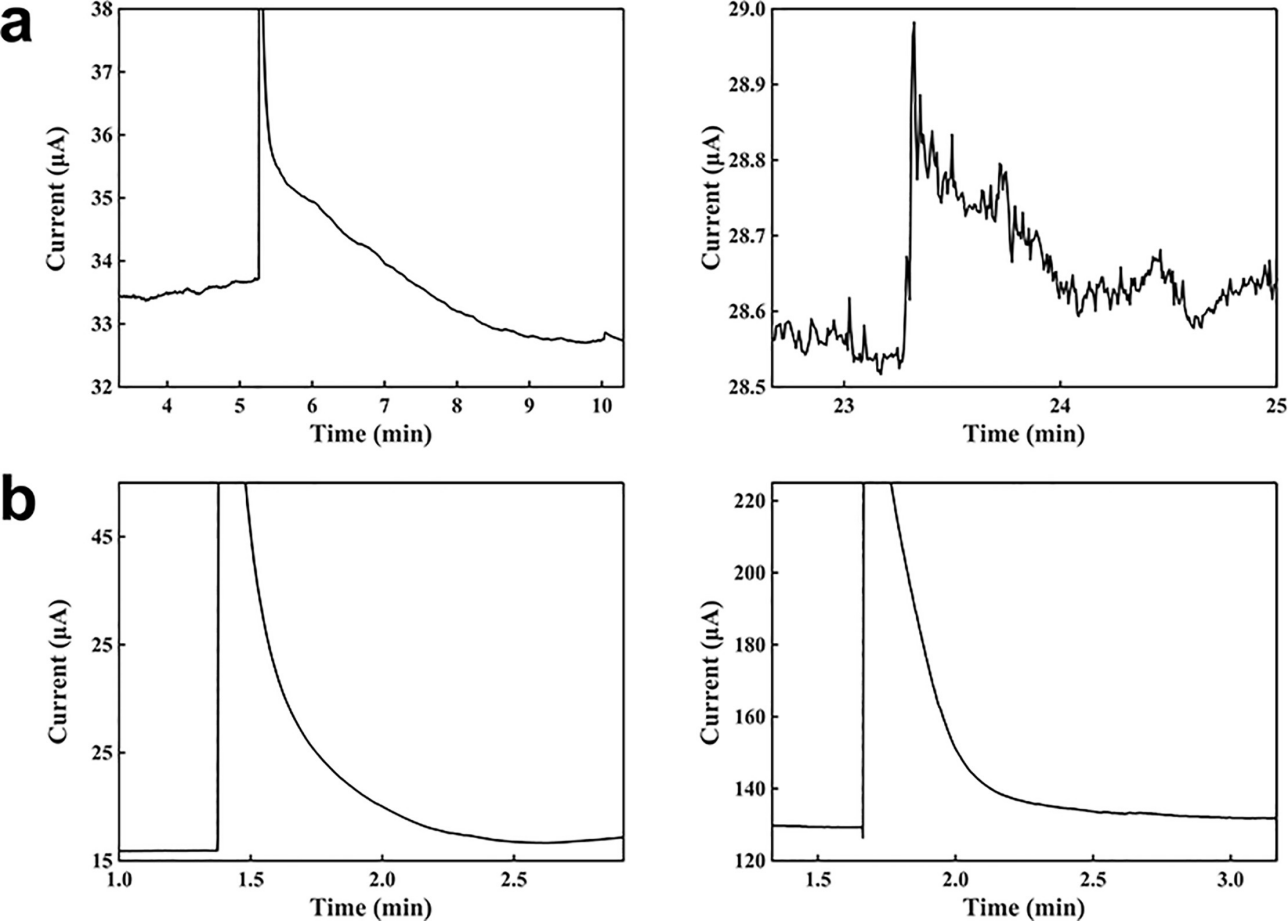
Study of a battery and a potentiostat for detecting H_2_O_2_ at different voltages. (a) Signal response curves for H_2_O_2_ using batteries at 1.5 V (left) and 3.0 V (right). (b) Signal response curves for H_2_O_2_ using a potentiostat at 1.5 V (left) and 3.0 V (right). Buffer solution: 50 mM. H_2_O_2_ concentration in the buffer: 0.05 mM.

Fig 2 shows a comparison of a potentiostat system and a battery system with different voltages. Fig 2A left shows the characterization of 1 mM H_2_O_2_ with a voltage of 1.5 V. After adding 100 μl of buffer solution to the sensor, 5 μl of 1 mM H_2_O_2_ was added to the buffer solution. The response time was about 4 min, and the sensitivity was -1.89 μA∙mM^-1^. The current increased rapidly after the addition of H_2_O_2_, due to the strong oxidation of H_2_O_2_ on the electrode surface. Then, the current decreased to be about the initial baseline, since the H_2_O_2_ molecules were oxidized rapidly with the high voltage from the battery, and all the H_2_O_2_ molecules were consumed after a while. Fig 2A right shows the current-versus-time response curve for the H_2_O_2_ detection with the sensor powered by a voltage of 3.0 V. Similarly, after adding 100 μl of buffer solution, 5 μl of 1 mM H_2_O_2_ was added. The response time was about 2 min, the sensitivity was 2.10 μA∙mM^-1^, and detection limit was 0.014286 mM. Fig 2B left shows the signal response curve for detecting H_2_O_2_ with a potentiostat at a constant voltage of 1.5 V. With the same experimental procedure described above, the response time was 2 min, the current increased by 0.78 μA, the sensitivity was 15.75 μA∙mM^-1^, and detection limit was 0.001905 mM. Fig 2B right shows the current-versus-time response plot of H_2_O_2_ using a potentiostat with a voltage of 3.0 V. The current baseline was 129.30000 μA, the response time was 2 min, the sensitivity was 194.04000 μA∙mM^-1^, and detection limit was 0.001546 mM. These results show that the sensitivity of the sensor with a battery was lower than that with a potentiostat, and the response times of these two methods were similar. Being powered by the batteries at 1.5 and 3.0 V, the sensor shows similar sensing performances. By contrast, as shown in Fig 2B, powered by the potentiostat at 1.5 and 3.0 V, the sensor shows clear different sensing performances; the current baseline with the 3.0 V was greatly higher, and the signal response was 12 times. Furthermore, for both of the potentiostat and battery, the supply voltage of 3.0 V resulted in a large noise and a larger instability, compared with the 1.5 V, owing to the higher current at the electrode and the larger amount of generated gas from the electrochemical reaction on the surface, thereby making the sensing system more unstable.

**Fig 3:**
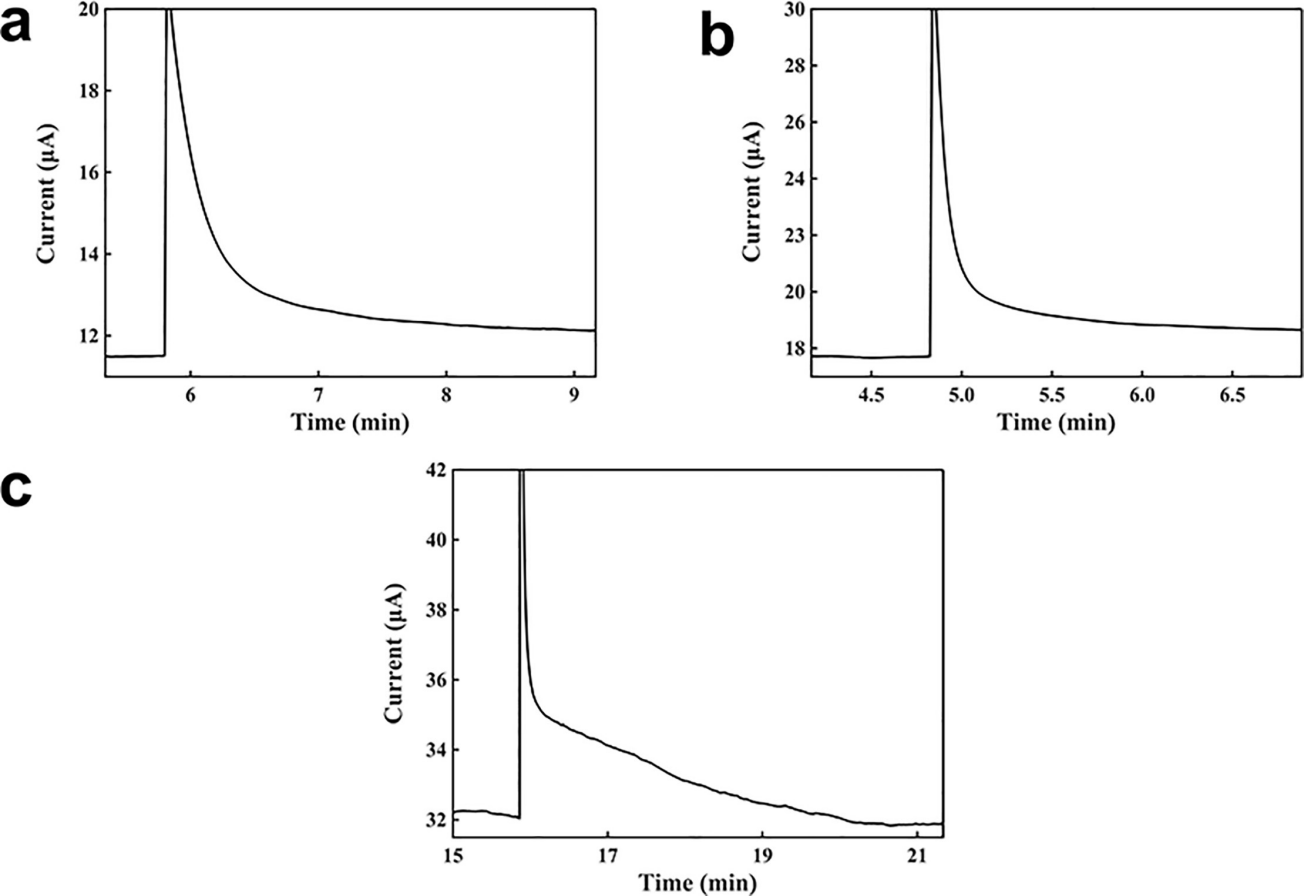
Study of different buffer concentrations with the battery powered sensor system. (a) A response curve for H_2_O_2_ with 0.5 mM buffer. (b) A response curve for H_2_O_2_ with 2 mM buffer. (c) A response curve for H_2_O_2_ with 50 mM buffer. H_2_O_2_ concentration in the buffer: 0.05 mM.

The buffer concentrations were studied to optimize the battery-powered sensing system, as shown in Fig 3. In this study, a 100 μl of the buffer solution was first placed on the sensor, and then 5 μl of 1 mM H_2_O_2_ was added to it; the mixture was left to stand until it reached a steady state. First, a 0.5 mM buffer solution was studied for detecting H_2_O_2_ using the battery-powered sensor, as shown in Fig 3A. The response time was about 3 min, the sensitivity was 3.7609 μA∙mM^-1^, the detection limit was 0.00638 mM, and current baseline was 11.4863 μA. Fig 3B shows the study of a 2.0 mM buffer solution for detecting H_2_O_2_. The response time was about 7 min, the sensitivity was 4.3138 μA∙mM^-1^, the current baseline was 17.797 μA, and detection limit was 0.00556 mM. Fig 3C shows the study of a 50.0 mM buffer solution detecting H_2_O_2_. The response time was 6 min, slope was -1.533 μA∙mM^-1^, and current baseline was 33.6964 μA. The current baseline increased as the buffer concentration increased, and a concentration of 50 mM resulted in the highest current baseline. For the detection of H_2_O_2_, the maximum signal response was obtained at a 2 mM buffer concentration. For the 2.0 mM buffer solution, the ion concentration was higher, and the electron transfer rate was larger, compared with the 0.5 mM buffer solution; in turn, the current response with the 2.0 mM was higher than that with the 0.5 mM. However, when the buffer concentration was 50 mM, the concentration was too high, and the current baseline was much larger than that with the 2.0 mM buffer. It may be because the H_2_O_2_ consumption rate and the electron density passing through the electrode increased dramatically, the H_2_O_2_ concentration was not stable in the droplet. Therefore, the current was not stabilized easily and the signal response was smaller.

### 3.3. Comparison of a potentiostat and a battery for H_2_O_2_ detection

**Fig 4:**
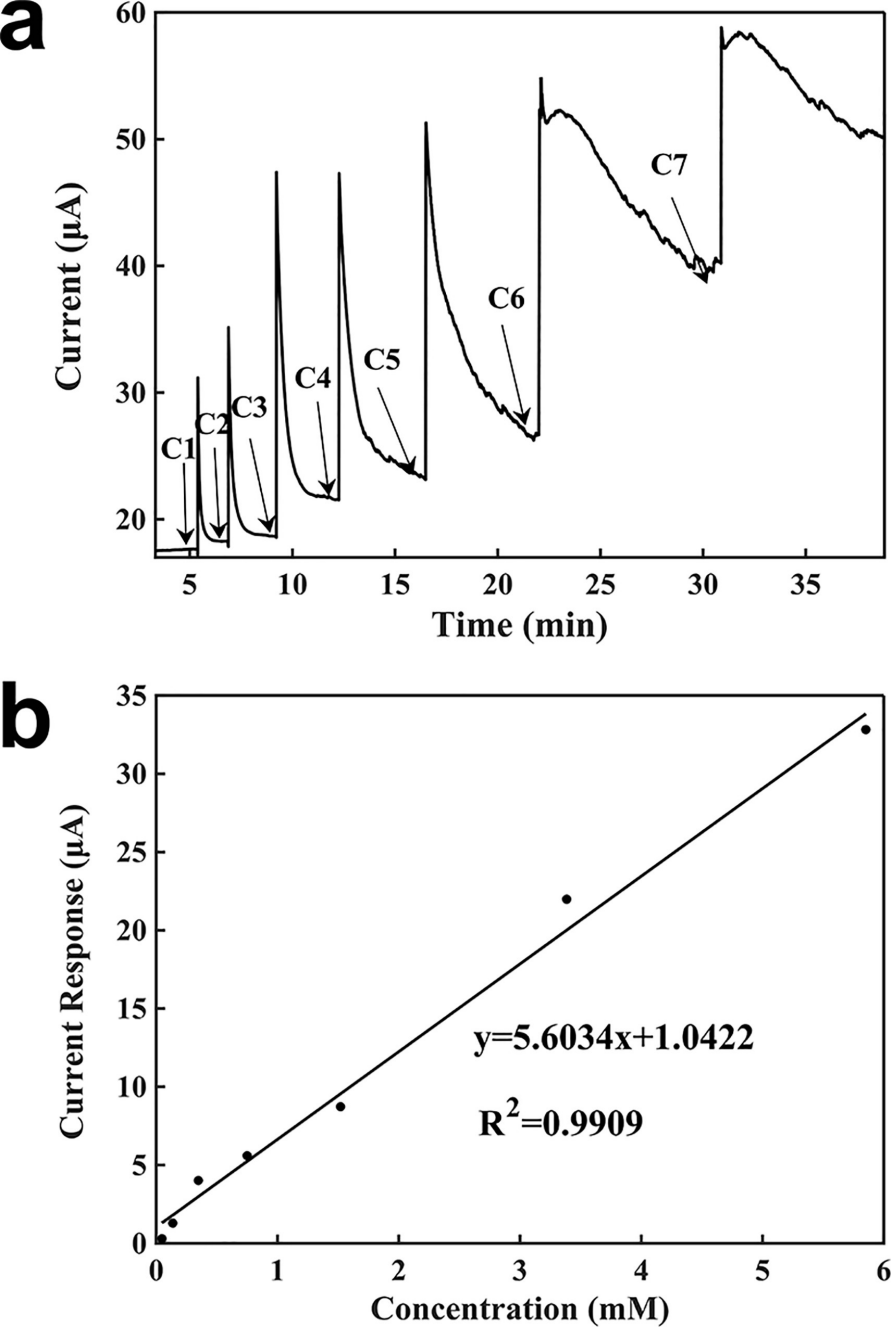
Characterization of the sensor for the detection of H_2_O_2_ using a potentiostat. (a) Current-verses-time response curve upon the additions of H_2_O_2_. C1: 0.04762mM, C2: 0.08875mM, C3: 0.21146mM, C4: 0.40217 mM, C5: 0.77000 mM, C6: 1.86462 mM, C7: 2.46724 mM. (b) Calibration curve for the detection of H_2_O_2_.

Fig 4 shows the characterization of H_2_O_2_ detection based on a graphite biosensor using the potentiostat. The potentiostat was used for measurement at voltage of 1.5 V. A 100 μl of 2 mM buffer solution was placed on the sensor and then 5 μl of each concentration of H_2_O_2_ solution was added; the mixture was left to stand until the current stabilized, and then, the next concentration of H_2_O_2_ was continuously added dropwise. Fig 4A shows the current-versus-time curve for H_2_O_2_ detection. The current baseline was 17.6500 μA. When the solution was added, the newly added H_2_O_2_ molecules were concentrated around the electrode surface. Then, a large number of the H_2_O_2_ molecules were made contact with the electrode surface, and a strong oxidation was occurred on the electrode surface. Therefore, a large current was generated. After this, the H_2_O_2_ molecules continue to diffuse rapidly, and the number of H_2_O_2_ molecules on the electrode surface was reduced. As the diffusion rate decreased gradually, the current reached a stable state, when the concentration became uniform in the droplet. Moreover, as the concentration in the droplet increased, the diffusion time and the measuring time increased, and it became more difficult for the current to reach equilibrium. The measuring time was about 5 min. From the calibration curve in Fig 4B, the slope was 5.6034 μA∙mM^-1^, the regression coefficient was 0.9909, and detection limit was 0.00535 mM.

**Fig 5:**
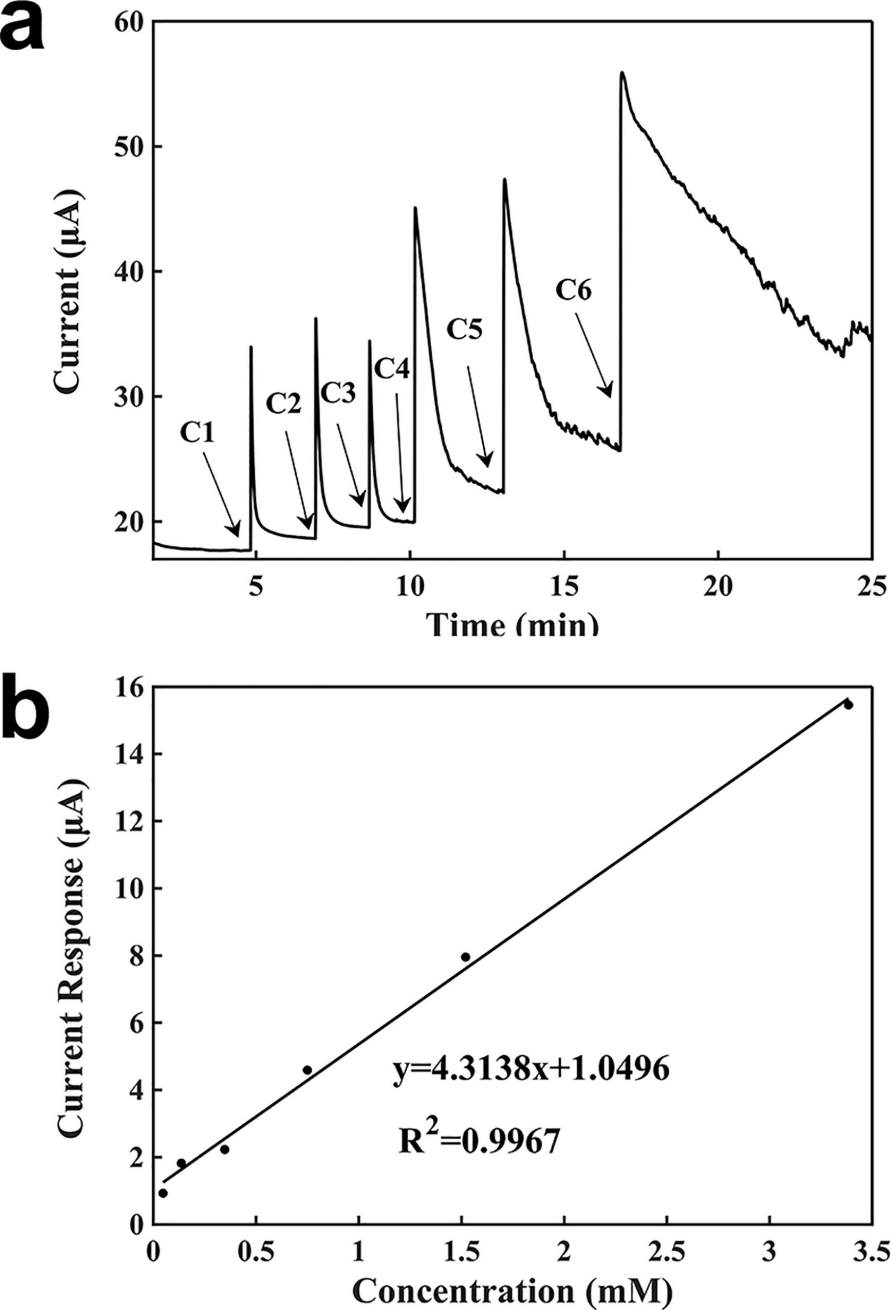
Characterization of the sensor for the detection of H_2_O_2_ with a battery. (a) Current-verses-time response curve upon the additions of H_2_O_2_. C1: 0.04762 mM, C2: 0.08875 mM, C3: 0.21146 mM, C4: 0.40217 mM, C5: 0.77000 mM, C6: 1.86462 mM. (b) Calibration curve for the detection of H_2_O_2_.

Fig 5 shows the characterization of H_2_O_2_ detection using a battery-powered graphite biosensor. A multimeter was used together with a button cell battery at 1.5 V for sensing. As shown in Fig 5A, the current baseline was 17.6715 μA. After adding 100 μl of 2 mM buffer solution, 5 μl of a concentration of H_2_O_2_ was added into the buffer droplet. When a H_2_O_2_ solution was added, it generated a large current. Then, the current decreased with the diffusion of the H_2_O_2_ molecules in the droplet, and it gradually reached equilibrium when the H_2_O_2_ concentration in the droplet became uniform. After the current becoming stable, another concentration of H_2_O_2_ was added. As the H_2_O_2_ concentration in the droplet increased, the response time also increased, and it took a longer time for the current to reach equilibrium. The measuring time was about 4 min. From the calibration curve in Fig 5B, the slope was 4.3138 μA∙mM^-1^, the regression coefficient was 0.9967, and detection limit was 0.00556 mM.

The differences in the sensing performances for the detection of H_2_O_2_ by using a potentiostat and a battery were negligible. The signal responses by using a potentiostat and a battery were almost the same. The detection limit and the slope of the potentiostat were slightly better.

### 3.4. Comparison of a potentiostat and a battery for lactate detection

**Fig 6:**
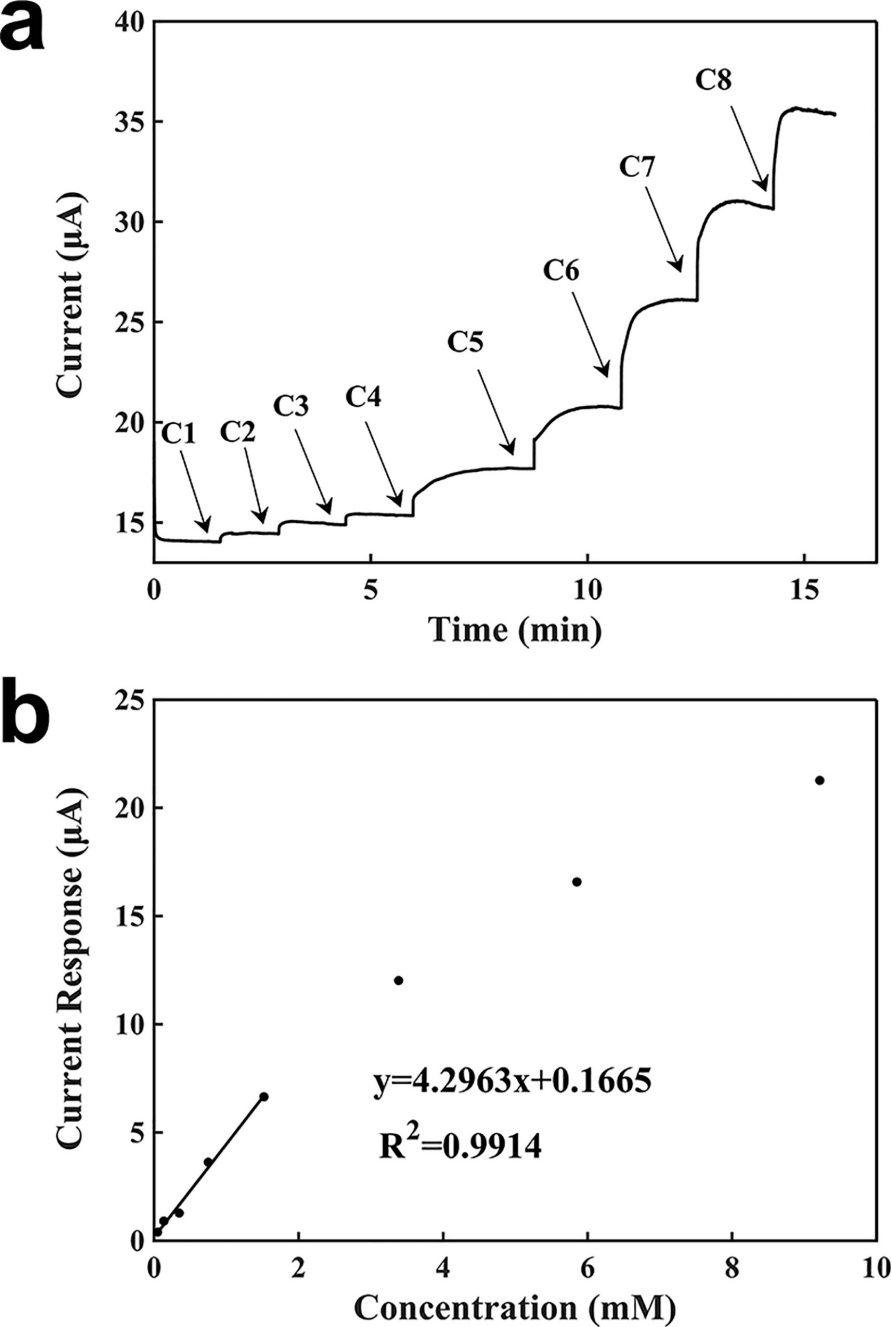
Characterization of the sensor for the detection of lactate using a potentiostat. (a) Current-verses-time response curve upon the additions of lactate. C1: 0.04762mM, C2: 0.08875mM, C3: 0.21146 mM, C4: 0.40217 mM, C5: 0.77000 mM, C6: 1.86462 mM, C7: 2.46724 mM, C8: 3.36243 mM. (b) Calibration curve for the detection of lactate.

Fig 6 shows the characterization of lactate detection based on a graphite biosensor using the potentiostat. The potentiostat was set to a voltage of 1.5 V. The current baseline was 14.0600 μA. Similarly, a 100 μl of 2 mM buffer solution was added onto the surface of the working electrode, followed by the addition of 5 μl of each concentration of H_2_O_2_ sequentially. When the lactate solution is adding, the lactate molecules were concentrated around the electrode surface. Because lactate took some time to produce H_2_O_2_, the electrical signal had a hysteresis time, and then it increased from the current baseline; this is different from the response signal of H_2_O_2_. The generation rate of lactate was greater than the sum of the diffusion and consumption rates of H_2_O_2_, and therefore, the signal rised slowly until these two rates were basically equal, at which time the current became stable. After the current being stable, the next concentration of lactate was added into the droplet. The measuring time with the potentiostat was 2 min. The generated signal response was proportional to the lactate concentration. From the calibration curve in Fig 6B, the resulting slope was 4.2963 μA∙mM^-1^, the regression coefficient was 0.9914, and detection limit was 0.00698 mM.

**Fig 7:**
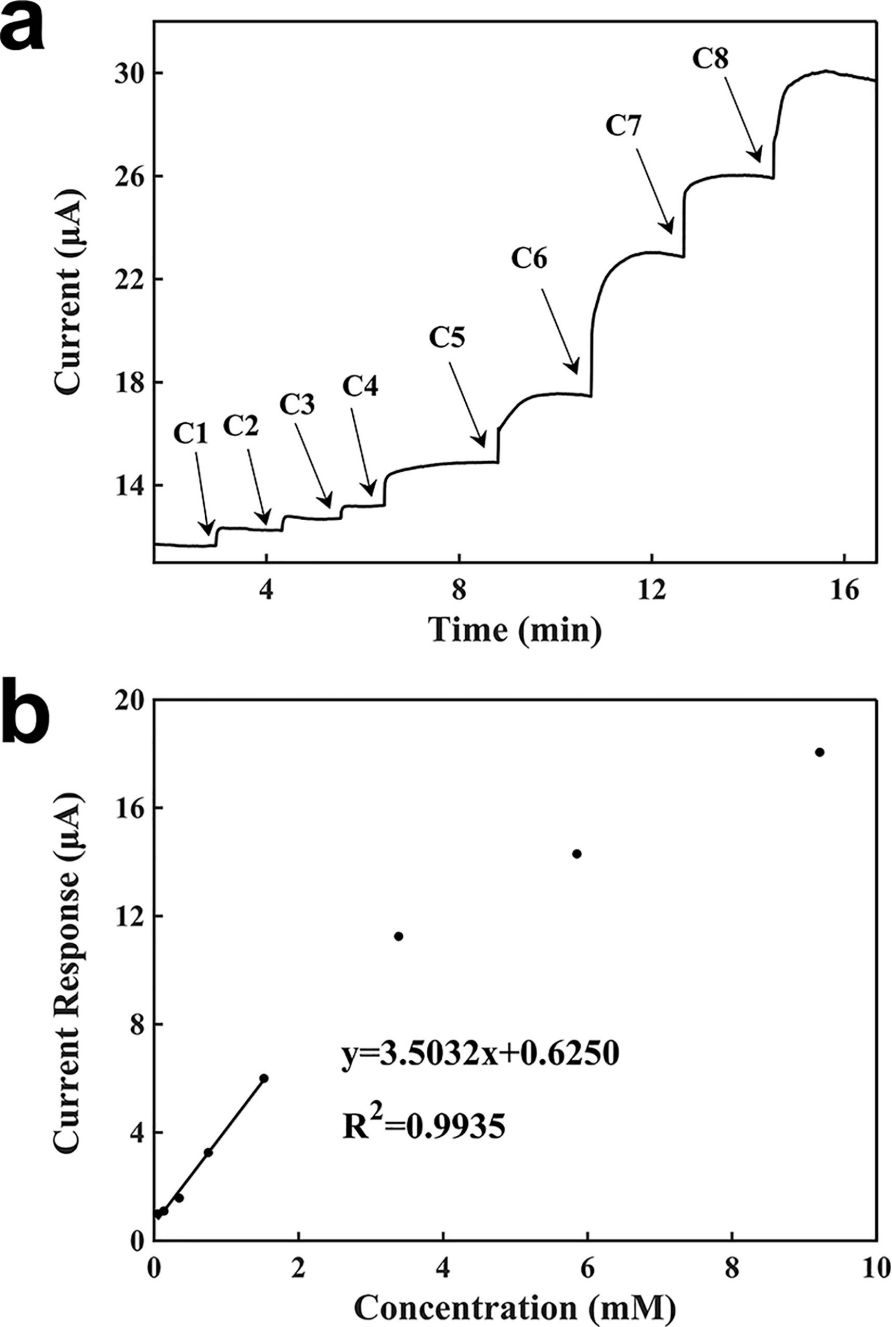
Characterization of the sensor for the detection of lactate using a battery. (a) Current-verses-time response curve upon the additions of lactate. C1: 0.04762 mM, C2: 0.08875 mM, C3: 0.21146 mM, C4: 0.40217 mM, C5: 0.77000 mM, C6: 1.86462 mM, C7: 2.46724 mM, C8: 3.36243 mM. (b) Calibration curve for the detection of lactate.

Fig 7 shows the characterization of lactate detection using a battery-powered graphite biosensor. A battery voltage of 1.5 V was used. As shown in Fig 7A, the current baseline was 11.6200 μA. 100 μl of 2 mM buffer was added, and 5 μl of each lactate concentration was added to the top of the working electrode sequentially. Similarly, when a lactate solution was added, the current became to increase until it reached a steady state. After the current becoming stable, another concentration of lactate was added. The measuring time with the battery-powered sensor was about 2 min. The signal response was proportional to the lactate concentration. The slope was 3.5032 μA∙mM^-1^, regression coefficient was 0.9935, and detection limit was 0.00771 mM.

The differences in the sensing performances for the detection of lactate by using a potentiostat and a battery were negligible. The detection limit, the slope of the calibration curve, and the detection time of the sensor powered by the potentiostat were slightly better.

## 4. Conclusion

In this study, we have developed a sensor system powered by a button battery and compared its performance with that of a traditional potentiostat for detecting lactate. We found that both systems showed similar sensing performances. By longitudinally comparing the effect of experimental conditions such as the buffer concentration and the voltage on the sensing performance, we verified that the battery-powered sensor shows an excellent detection performance, making it a promising alternative for electrochemical measurements in the future. The battery-powered sensor is greatly simplified in size and structure relative to the potentiostat, allowing us to mount it on items such as a little ring to detect the characteristics of sweat ubiquitously. We expect that this study could open up significant avenues for developing new sensing systems and advance related biomedical and environmental applications.
